# Cardiogenic shock managed with phlebotomy: an unusual case of end-stage cardiac hemochromatosis

**DOI:** 10.1093/ehjcr/ytag087

**Published:** 2026-02-06

**Authors:** Zoha Majeed, J Emanuel Finet, Mazen Hanna, Deborah Kwon, Arianne Clare Agdamag

**Affiliations:** Heart, Vascular and Thoracic Institute (HVTI), Cleveland Clinic, 9500 Euclid Avenue, Cleveland, OH 44195, USA; Heart, Vascular and Thoracic Institute (HVTI), Cleveland Clinic, 9500 Euclid Avenue, Cleveland, OH 44195, USA; Heart, Vascular and Thoracic Institute (HVTI), Cleveland Clinic, 9500 Euclid Avenue, Cleveland, OH 44195, USA; Heart, Vascular and Thoracic Institute (HVTI), Cleveland Clinic, 9500 Euclid Avenue, Cleveland, OH 44195, USA; Heart, Vascular and Thoracic Institute (HVTI), Cleveland Clinic, 9500 Euclid Avenue, Cleveland, OH 44195, USA

**Keywords:** Hereditary Hemochromatosis, Heart Failure, Chelation, Therapeutic Phlebotomy, Case report, Infiltrative Cardiomyopathy

## Abstract

**Background:**

Iron-overload cardiomyopathies can be a diagnostic challenge and are often overlooked in patients with new-onset heart failure with systolic and diastolic dysfunction. They can present as primary hemochromatosis, characterized by HFE gene mutations causing abnormal iron sensing and subsequent storage in various organs of the body, or as secondary overload syndromes in patients with history of transfusions. We present a case of a patient with end-stage hereditary hemochromatosis treated with phlebotomy and chelation therapy that had improvement in systolic function on follow-up.

**Case:**

The patient is a 63-year-old female with new onset heart failure with systolic and diastolic dysfunction who presented for evaluation to our clinic with signs and symptoms of decompensated heart failure. She was admitted for management and further work up revealed hemochromatosis with multiple organ system involvement. Treatment was initiated with phlebotomy and chelation therapy. Follow-up echocardiogram revealed significant improvement in systolic and diastolic dysfunction no longer necessitating transplant work up.

**Discussion:**

Iron-overload syndromes are often asymptomatic early in the disease with evidence of rapid deterioration once there is clinical evidence of heart failure. Therapeutic phlebotomy is the treatment of choice in non-anemic patients before severe complications including cardiomyopathy develops. Our clinical case highlights a significant improvement in hemodynamics after initiation of phlebotomy. Phlebotomy is beneficial because it depletes body iron stores and oxidative stress and enhances vascular function.

Learning points

- To highlight the importance of testing for hemochromatosis as a cause of new onset systolic and diastolic heart failure.
- To illustrate the role of phlebotomy and chelation therapy in cardiogenic shock in patients with hemochromatosis.
- To prioritize close follow-up on discharge and early consideration for heart transplant.

## Introduction

Hereditary hemochromatosis (HH) comprises a group of genetic disorders characterized by dysregulated iron sensing and increased intestinal absorption, leading to progressive iron overload.^1^ Excess iron accumulates in multiple organs, including the liver, joints, endocrine glands, and heart, resulting in progressive dysfunction.^2^ Cardiac involvement arises from deposition of ferrous iron (Fe²⁺) within cardiomyocytes, promoting the formation of reactive oxygen species (ROS) and driving cellular injury, fibrosis, arrhythmias, and ultimately heart failure.^3,4^ Early recognition and treatment of hemochromatosis are essential to prevent irreversible organ damage and improve outcomes.

## Summary figure

**Figure:**
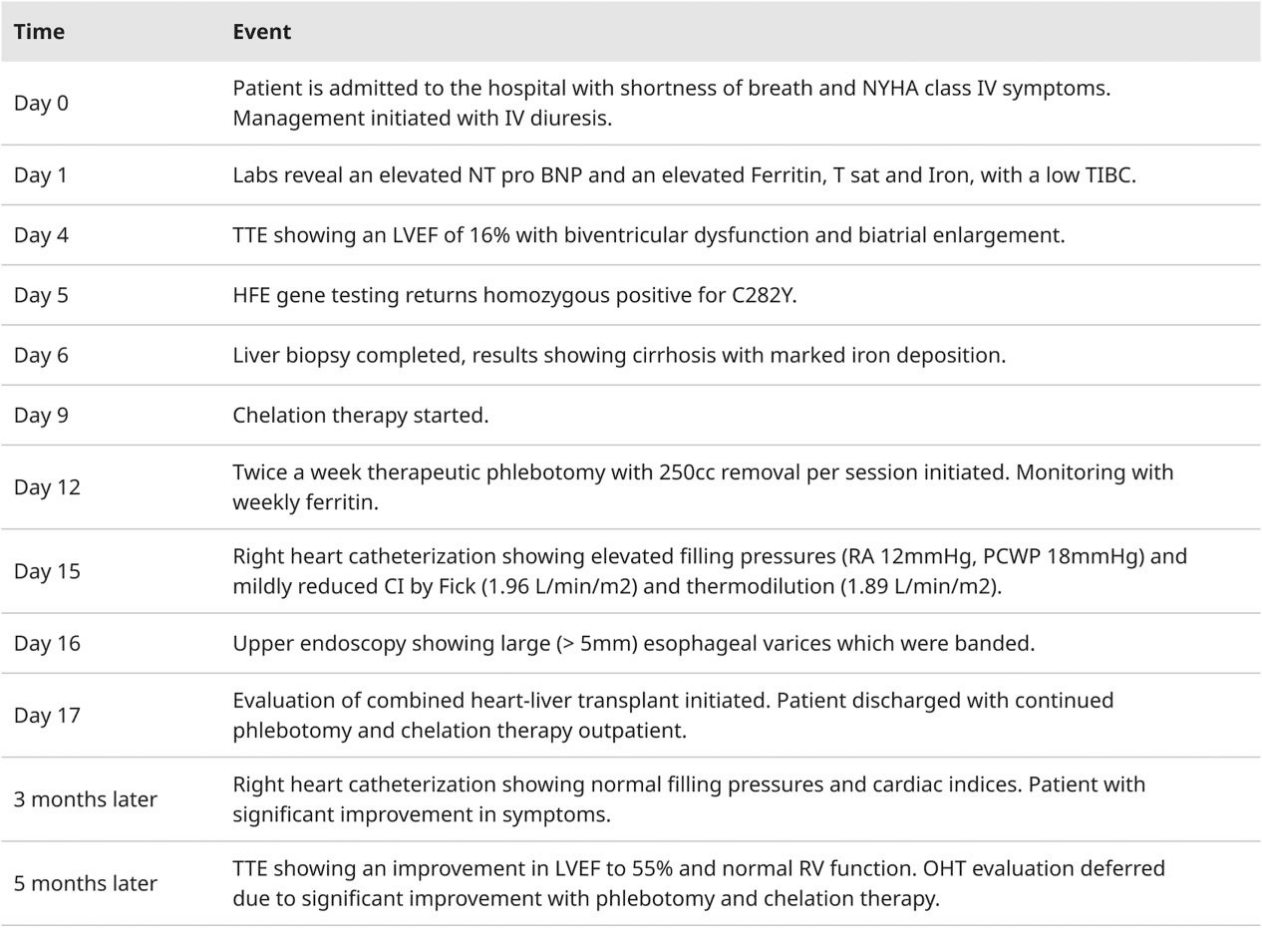


## Case presentation

A 63-year-old woman with a history of heart failure with reduced ejection fraction (HFrEF) presented with worsening dyspnoea, orthopnea, peripheral oedema, and abdominal distention. A prior nuclear myocardial perfusion imaging study demonstrated no evidence of ischaemia, supporting a non-ischemic cardiomyopathy (NICM).

She had experienced multiple admissions for acute decompensated heart failure and was unable to tolerate optimal doses of guideline-directed medical therapy (GDMT) due to symptomatic hypotension. Her past medical history included breast cancer treated with mastectomy, chemotherapy, and radiotherapy in 1998, as well as chronic obstructive pulmonary disease (COPD) requiring home oxygen. Her outpatient medications included spironolactone, empagliflozin, and midodrine. A recent echocardiogram showed a left ventricular ejection fraction (LVEF) of 15%–20% with severe global hypokinesis, moderate right ventricular (RV) dilation, and moderate tricuspid regurgitation (TR).

She was admitted with New York Heart Association (NYHA) class IV symptoms and stage D heart failure. NT-proBNP was elevated at 4498 pg/mL (reference <125 pg/mL), and she was started on cautious intravenous diuresis followed by positive inotropic support with digoxin. A repeat echocardiogram demonstrated an LVEF of 16%, global hypokinesis, moderate left ventricular dilation, and RV dysfunction (see Supplementary material online, *Videos S1*–*S7*). A systematic evaluation for non-ischemic aetiologies of HFrEF was pursued in tandem.

Electrocardiography showed normal sinus rhythm with a known right bundle branch block. Alcoholic cardiomyopathy was excluded based on history. Given her remote history of chemotherapy and chest radiotherapy for breast cancer, chemotherapy-related cardiotoxicity was considered; however, treatment records were unavailable for review. Biventricular dilation with global hypokinesis raised concern for an infiltrative cardiomyopathy, with differential considerations including cardiac amyloidosis, sarcoidosis, and iron-overload cardiomyopathy.

Iron studies obtained at admission showed serum iron 199 μg/dL (reference 60–170 μg/dL), ferritin 7330 ng/mL (reference 13–150 ng/mL), and transferrin saturation 87.7% (reference 20%–45%), strongly suggesting iron-overload cardiomyopathy. Right heart catheterization showed elevated biventricular filling pressures (right atrial pressure 12 mmHg, pulmonary capillary wedge pressure 18 mmHg), combined pre- and post-capillary pulmonary hypertension, and a low cardiac index of 1.96 L/min/m² by estimated Fick—consistent with a low-output cardiac state (*Table 1*).

**Table 1:** Pre- and post-phlebotomy right heart catheterization hemodynamics

Variable	Pre-phlebotomy	Post-phlebotomy
RA mean	12 mmHg	2 mmHg
RV pressure	50 mmHg/14 mmHg	30 mmHg/2 mmHg
PA pressure (mean)	50 mmHg/22 mmHg (32)	26 mmHg/9 mmHg (15)
PCWP mean	18 mmHg	7 mmHg
Fick CO, CI	3.10 L/min, 1.96 L/min/m^2^	4.45 L/min, 3.07 L/min/m^2^
Thermodilution CO/CI	3.02 L/min, 1.89 L/min/m^2^	3.97 L/min, 2.74 L/min/m^2^

This table shows the pre-treatment and post-treatment right heart catheterization values indicating a significant improvement in cardiac indices and reduction in biventricular filling pressures.

**Figure. 1:**
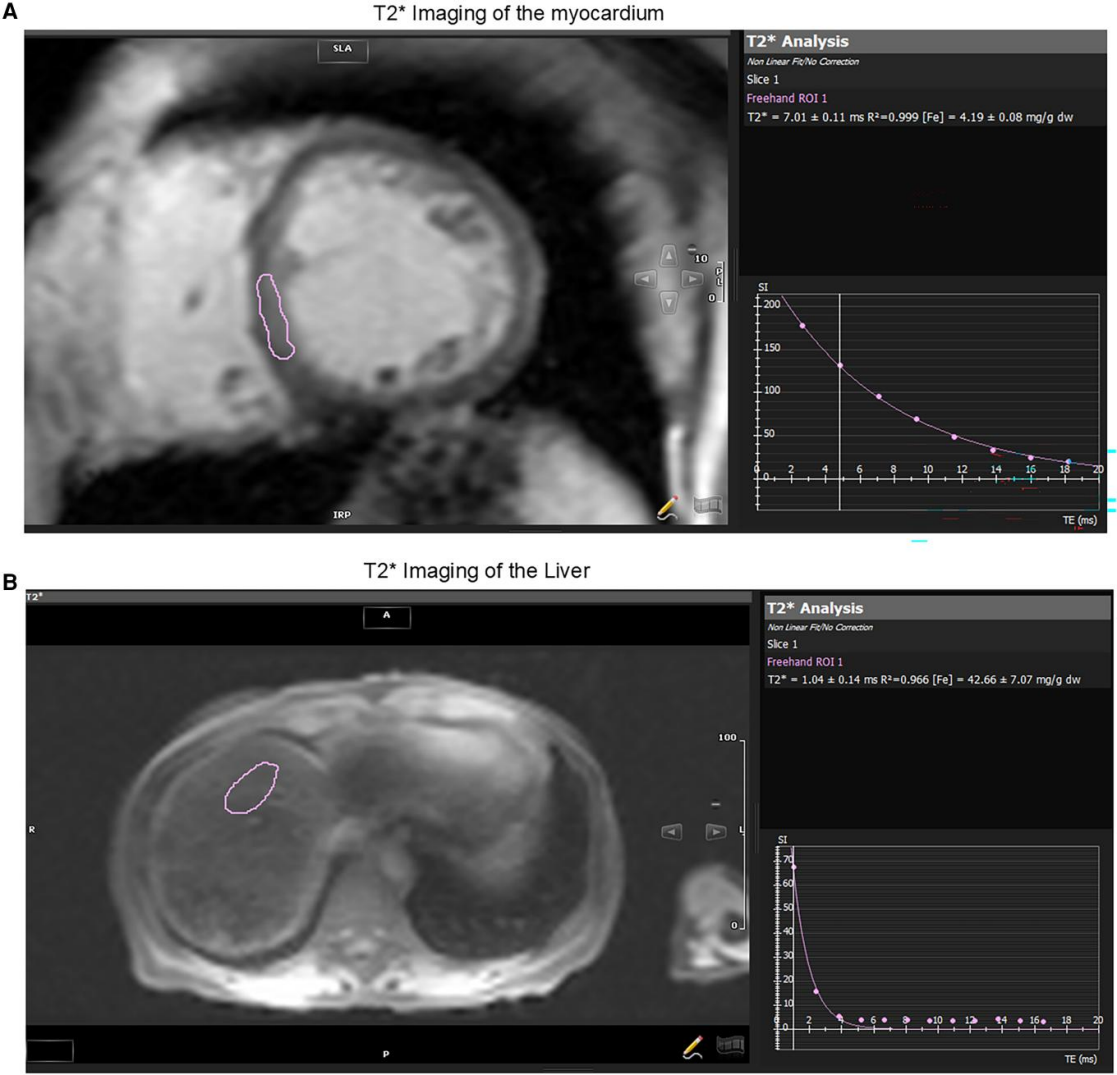
(*A*) T2* imaging for the cardiac myocardium showing a T2* of 7.0 ms, (normal >20 ms; <10 ms indicating severe iron deposition) diagnostic of cardiac hemochromatosis. (*B*) T2* imaging of the liver showing a T2* of 1.0 ms, (normal >6 ms, <3mc indicating severe iron deposition) diagnostic of liver hemochromatosis.

**Figure. 2:**
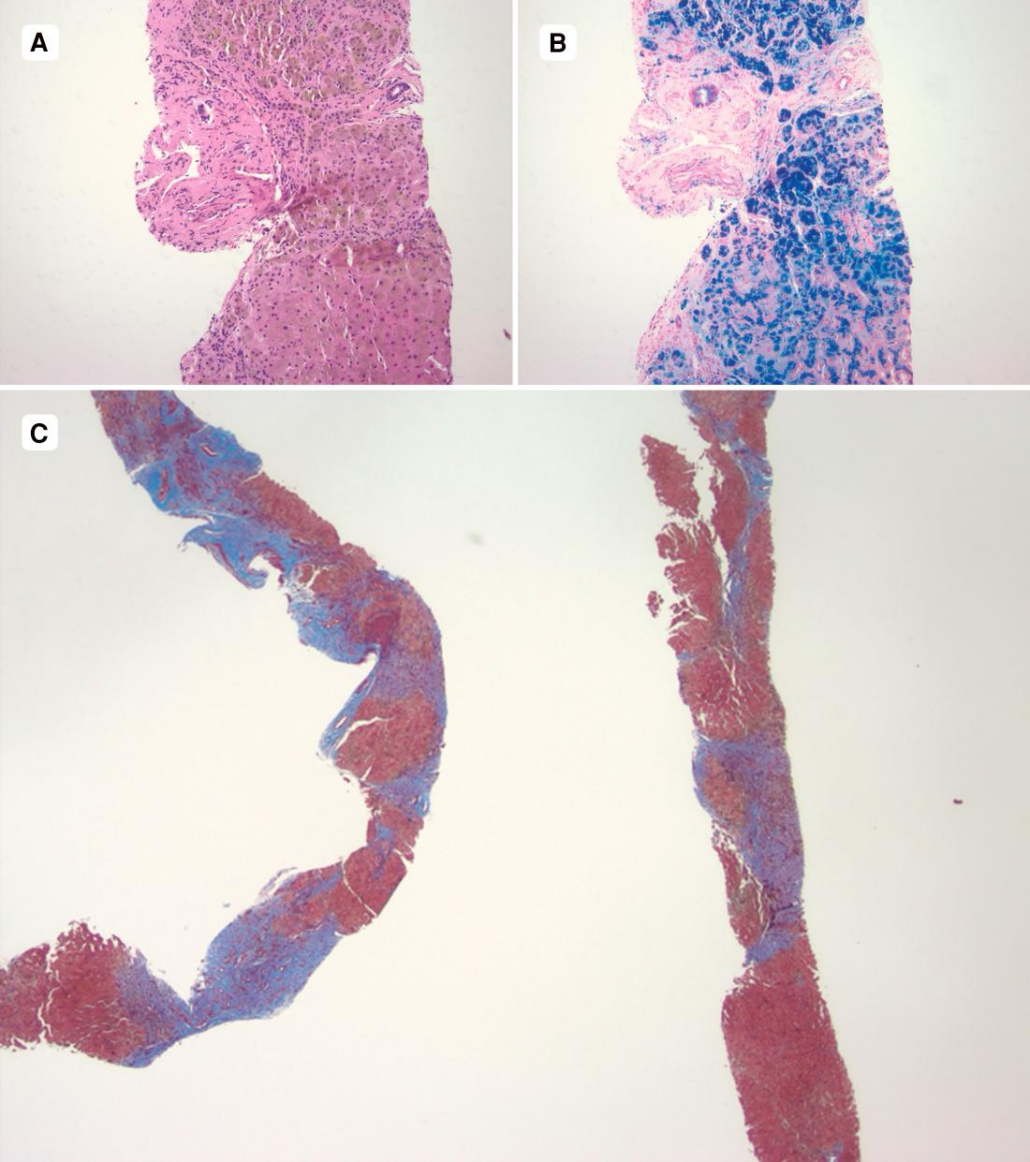
(*A*) Hepatocytes and biliary epithelium contain granular brown pigmented material compatible with hemosiderin (H&E stain, 10× magnification). (*B*) Hemosiderin in hepatocytes and biliary epithelium shows blue staining on iron stain (iron stain, 10×). (*C*) Trichrome stain shows Diffuse bridging fibrosis and regenerative nodule formation compatible with cirrhosis on trichrome stain. The trichrome stain shows red staining of hepatocytes and blue staining of fibrosis.

In consultation with haematology and hepatology, cardiac magnetic resonance imaging (CMR) was pursued, which demonstrated reduced LVEF with a myocardial T2* of 7.0 ms (normal >20 ms; <10 ms indicating severe iron deposition) and a hepatic T2* of 1.0 ms (normal >6 ms; <3 ms indicating severe iron overload), confirming multiorgan iron overload (*Figure 1*). Subsequent liver biopsy revealed marked hepatic iron accumulation with cirrhosis, and upper endoscopy demonstrated large oesophageal varices requiring band ligation (*Figure 2*). Given the evidence of multiorgan involvement, the patient was referred for evaluation for combined heart–liver transplantation.

Treatment with therapeutic phlebotomy (250 mL twice-weekly) and intravenous deferoxamine was initiated as part of a multidisciplinary management strategy for suspected HH. HFE genetic testing obtained for etiologic confirmation later demonstrated homozygous C282Y mutation, establishing the diagnosis. She was started on low-dose beta-blocker therapy, mineralocorticoid receptor antagonist (MRA), and SGLT2 inhibitor. She was discharged with scheduled twice-weekly phlebotomy and ongoing chelation therapy with close outpatient follow-up.

Over the following months, she experienced significant functional improvement without further heart failure admissions. Blood work showed improvement in ferritin to 1933 ng/mL, NT-proBNP normalized to 116 pg/mL, and a repeat echocardiogram at five months post-treatment confirmed normalization of LVEF. Her transplant evaluation was subsequently closed.

## Discussion

Hepcidin is the key regulator of iron homeostasis, controlling uptake from enterocytes and macrophages. Deficient hepcidin expression underlies most forms of HH, leading to excessive iron absorption and deposition in multiple organs.^5^ Iron overload is often clinically silent until end-organ dysfunction emerges. Early cardiac manifestations include diastolic dysfunction and concentric remodelling, progressing to dilated cardiomyopathy with systolic failure and arrhythmias conferring a poor prognosis.^6^ Early diagnosis and intervention are therefore essential, even in asymptomatic individuals.

Diagnostic criteria for hemochromatosis include a ferritin of >200 ng/mL and a transferrin saturation of more than 45% for premenopausal women, and a ferritin > 300 and a transferrin saturation >50% for males and post-menopausal women. While ferritin is an acute phase reactant, unexplained hyperferritinemia with elevated transferrin saturation warrants HFE gene testing.^2^

Therapeutic phlebotomy is the treatment of choice in non-anemic patients before severe complications, including cardiomyopathy, develop. In patients with severe cardiomyopathy and end-stage heart failure, medical therapy in combination with phlebotomy and iron chelation can be utilized but have not consistently been shown benefit.^1^

Our clinical case highlights significant improvement in hemodynamics after initiation of phlebotomy and chelation therapy, suggesting a therapeutic benefit, as demonstrated by pre- and post-treatment right heart catheterizations (*Table. 1*). Phlebotomy leads to depletion of body iron stores which reduces oxidative stress and enhances vascular function. Phlebotomy reduces haematocrit thereby reducing plasma viscosity which is the major regulatory factor of flow resistance in the microcirculation and capillary perfusion.

This case also highlights the important role of blood rheology in iron-overload cardiomyopathy. Elevated blood viscosity increases cardiac workload and systemic vascular resistance, contributing to structural remodelling, elevated filling pressures, and chamber dilation.^7,8^ In this patient, these haemodynamic abnormalities were consistent with cardiogenic shock physiology, meeting criteria for SCAI stage C–D shock.

According to the European Society of Cardiology (ESC), cardiogenic shock is defined by sustained hypotension (systolic blood pressure <90 mmHg), evidence of end-organ hypoperfusion, and primary cardiac dysfunction.^9^ Landmark trials such as SHOCK and IABP-SHOCK II similarly characterize cardiogenic shock by hypotension, elevated filling pressures, reduced cardiac output, and signs of tissue hypoperfusion.^10,11^ These definitions help contextualize the severity of her presentation and highlight the importance of promptly identifying reversible causes.

In this patient, the combined use of GDMT and therapeutic phlebotomy with chelation therapy led to substantial improvement in biventricular cardiac function. Our patient demonstrated an improvement in LVEF from a pre-treatment value of 16% to a post-treatment value of 55%.^12^ (*Table. 2*) As part of her advanced heart failure management, she was also evaluated for combined heart–liver transplantation given the presence of both end-stage cardiac and hepatic dysfunction; however, this option was ultimately deferred due to clinical improvement and comorbid pulmonary disease.

**Table 2:** Pre- and post-phlebotomy echocardiogram findings

Variable	Pre-phlebotomy	Post-phlebotomy
LVEF	16%	55%
LV end diastolic volume	116 mL	62 mL
LV end systolic volume	98 mL	28 mL
LV stroke volume	18 mL	34 mL
Right ventricle systolic function	Severely decreased	Normal
Right ventricle	Dilated	Normal
Right atrium	Dilated	Normal

This table shows the pre-treatment and post-treatment echocardiogram findings, indicating a significant improvement in left ventricular ejection fraction and left and right ventricular stroke volume.

Our case suggests that early and effective management of hemochromatosis with phlebotomy, chelation therapy, GDMT and close outpatient follow-up as recommended by the ESC guidelines, can successfully improve cardiac function and lead to clinical improvement.^13^ It also emphasizes the importance of a multidisciplinary approach in managing complex cardiac cases and highlights the need to maintain a high index of suspicion for infiltrative cardiomyopathies, particularly in patients presenting with unexplained heart failure.

## Lead author biography


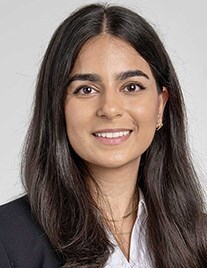


Dr. Zoha Majeed is an Internal Medicine Resident at the Cleveland Clinic with an interest in cardiovascular disease, particularly advanced heart failure and transplant. She completed her medical school in Lahore, Pakistan and moved to the US for further training. Her areas of focus include invasive hemodynamics, infiltrative cardiomyopathies, and the use of multimodal imaging techniques to aid in their diagnosis and management. She is passionate about combining clinical care with a deeper understanding of complex cardiovascular pathophysiology. In her free time, she enjoys hiking and experimenting with new recipes.

## Supplementary Material

ytag087_Supplementary_Data

## Data Availability

All data underlying this case are included in the article. No additional datasets are available.
